# Hydrogeochemical Characterization and Suitability Assessment of Groundwater: A Case Study in Central Sindh, Pakistan

**DOI:** 10.3390/ijerph16050886

**Published:** 2019-03-11

**Authors:** Muhammad Afnan Talib, Zhonghua Tang, Asfandyar Shahab, Jamil Siddique, Muhammad Faheem, Mehak Fatima

**Affiliations:** 1 School of Environmental Studies, China University of Geosciences, Wuhan 430074, China; afnantalib@cug.edu.cn (M.A.T.); jamils@qau.edu.pk (J.S.); faheem2u12@yahoo.com (M.F.); 2 College of Environmental Science & Engineering, Guilin University of Technology, Guilin 541000, China; Asfand_4u87@yahoo.com; 3 Department of Dermatology, University of Health Sciences, Lahore 54000, Pakistan; mehakfatima301@gmail.com

**Keywords:** groundwater pollution, water quality, hydrogeochemical modeling, Water Quality Index, central Sindh, Rohri Canal Command

## Abstract

Groundwater is the most important water resource, on which depends human geo-economic development and survival. Recent environmental changes and anthropogenic activities render groundwater severely vulnerable. Groundwater in Central Sindh, Pakistan, is facing a similar situation. Hydrogeochemical characteristics of the groundwater in the said region were investigated by analyzing 59 groundwater samples via agricultural and drinking indices, using various statistical methods and graphical approaches to identify factors affecting groundwater. Major reactions occurring in the groundwater system were quantified by hydrogeochemical modeling. A statistical summary reveals the abundance of cations is Na^+^ > Ca^2+^ > Mg^2+^ > K^+^, while the abundance of anions is HCO_3_^−^ > Cl^−^ > SO_4_^2^. Groundwater chemistry is mainly of rock dominance. Correlation analysis and graphical relationships between ions reveal that ion exchange and rock weathering such as the dissolution of halite, albite, and dissolution of carbonate minerals are important rock–water interactions, governing the evolution of groundwater chemistry. Hydrochemical facies are predominantly of mixed CaMgCl and Na-Cl type, with few samples of Ca-HCO_3_ type, which constitutes fresh recharged water. Based on the Water Quality Index (WQI), 28.82% samples were found to be unsuitable for drinking. A United States Salinity Laboratory (USSL) diagram, Wilcox diagram, and other agricultural indices indicate that majority of the groundwater samples fall within the acceptable range for irrigation purposes.

## 1. Introduction

The importance of freshwater resources and its provision for every form of life is inevitable [1]. Being the elixir of life, water resources ensure the stability of the ecosystem and the status of human health [2]. Out of the total volume of water on Earth (i.e., 1600 × 10^6^ km^3^), approximately 3.0 × 10^6^ km^3^ of groundwater and 0.1 × 10^6^ km^3^ of surface water is available as freshwater resources, which is 0.1938% of the total volume of water [3]. These approximations compel us to take strict initiatives for preservation of the available water resources to ensure its continuous and adequate availability for all living beings [4]. The key risks to freshwater resources are over-exploitation and over-consumption [5]. Since water scarcity is prevalent around the globe [6,7], groundwater exploitation emerges as an alternative to fulfill elevating water demands [8,9].

In the past few decades, global climatic change [10], urbanization, economic development [11], rapid population growth, and intensive industrialization renders the groundwater vulnerable and eventually deteriorating its quality, consequently risking environmental sustainability and preservation of life [12]. Groundwater pollution adversely affects the aqueous and terrestrial ecosystem [13], causing severe damage to human health [14,15]. Millions of people lose their lives being deprived of potable water [16] and using contaminated water [17]. Both anthropogenic and natural sources are posing significant threats to the quality of groundwater [18]. The use of pesticides, insecticides and fertilizers [19], waste from mining activities [20], industrial effluents [21], over-pumping [22], and interruptions in the river and canal networks are the prominent factors attributed to the anthropogenic activities. Moreover, natural processes include dissolution of rocks [23] and evaporation—particularly in shallow aquifers [24], which leads to water table rise and salt deposition, and seawater intrusion [25]. Considering all the adverse effects, it becomes vital to focus on groundwater management and protection from further deterioration.

Pakistan is a water-stressed country [26], not only facing a severe shortage of water [27] but is also threatened by its deteriorating quality. An abrupt decrease in both surface and groundwater resources has been noticed in the past few decades [28]. Out of the total annual water flows, Pakistan can only store 10%, which is only sufficient to meet the demand of the country for 30 days [29]. There has also been a drop in per capita availability of water from 5260 cubic meters in 1951 to 908 cubic meters in 2017, marking a significant drop of 82.73%. By 2025, with a population growth rate of 2.5% per annum, an additional population of 120 million will have to be fed. Hence, groundwater in Pakistan is mostly used for agricultural purposes besides being used for domestic and industrial purposes as well. Consequently, the demand of groundwater for domestic purposes would increase from 5.20 million acre feet to 9.70 million acre feet [30]. This drop is primarily credited to rapid population growth [31].

Sindh is the second most populated province in Pakistan. Physiographically, Sindh is bounded by the Kirthar and Laki ranges on the western side, Thar and cholistan deserts on eastern, and the Arabian sea to the south. The central part is extended from north-east to south-west, dominated by the lower Indus plain, Indus piedmont plain and Indus deltaic plain [32]. The available groundwater resource in Sindh is about 5 million acre foot (MAF) and has ample potential for irrigation however, the use of groundwater is comparatively lesser (4.3 billion cubic meter) than surface water because of two primary reasons: firstly, most of the area is lying on saline or brackish water; secondly, canal command areas are being provided with surface irrigation supplies [33]. The river Indus, having an influent behavior, loses water to the underlying aquifer, as it lies on a slight ridge, which slopes away in a lateral direction up to Larkana District. A part of the flow drains towards the desert in the east, whereas another flows towards the Khirthar Hills. During the harvest season of winter crops, the flow in the river below Sukkur Barrage becomes negligible, so the river starts receiving groundwater, especially from the left bank [33]. Studies have revealed contamination of groundwater by a variety of contaminants in Sindh province. Some of the water bodies are severely deteriorated by microbial contamination [34]; 53% of the area is affected by the calamitous outcomes of salinity and water-logging [35]; excessive fluoride exceeding the World Health Organization (WHO) and local thresholds has been reported in Nagar Parkar area [36]; and recent physiochemical analyses reveal elevated arsenic concentration in Matiari, Khairpur and Jamshoro districts [37]. Multiple approaches in the past few decades have been employed to address the hydrogeochemical characterization and groundwater quality assessment for drinking and irrigation purposes [38,39,40,41].

Categorically, the lower Indus plain comprises 14 cultivatable irrigation areas. The situation in the central Sindh, Rohri canal command area is vulnerable to surface and groundwater contamination which would ultimately risk the potability and agricultural utilization of groundwater. The literature regarding the evaluation of water quality in the study area is scarce. The present research will be explicitly helpful for the identification of hydrogeochemical characteristics and the processes governing the evolution of groundwater, and quality assessment for domestic and irrigation purposes. The findings may facilitate a clear understanding to address the adverse effects, and the solution is advocating the groundwater quality at canal command level in Sindh province.

## 2. Description of the Study Area

### 2.1. Location and Climate

**Figure 1:**
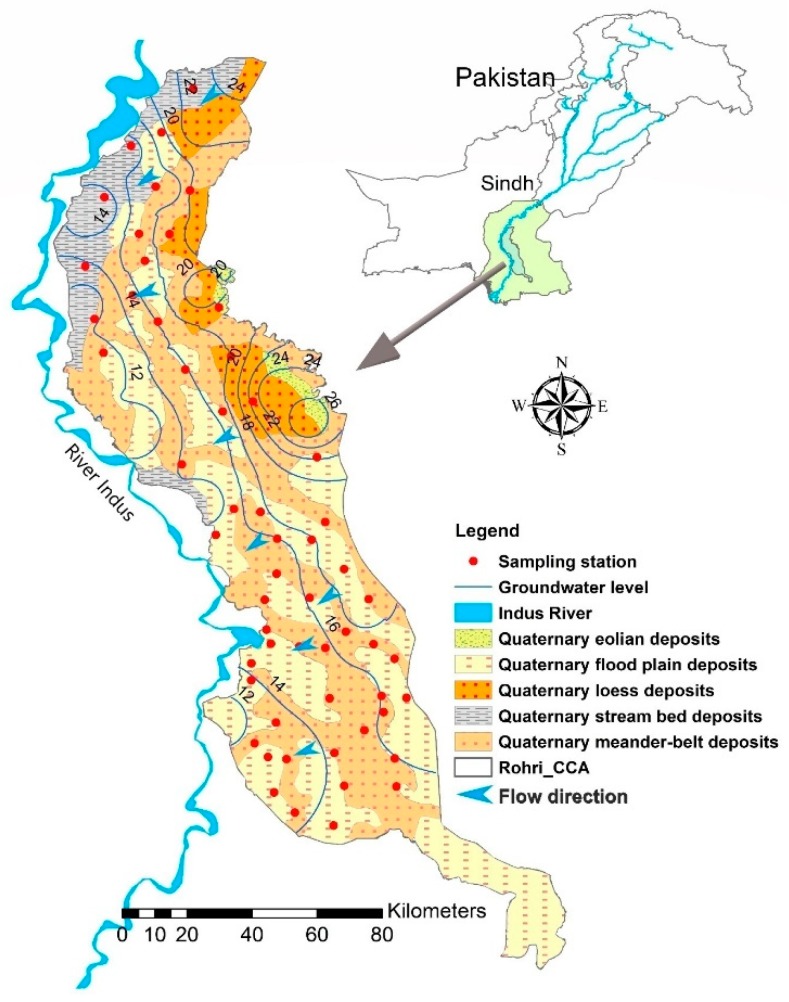
Location of the study area and sampling stations (Rohri Canal Command).

The study area (Rohri canal command area) is located in the middle of the lower Indus plain and is one of the 14 annuls commonly called Canal Command Areas [42]. It stretches from longitudes 67°54′ to 69°20′ E and latitudes 24°44′ N to 27°16′ N covering an area of about 11,639 km^2^ (Figure 1). The climate of the area is mostly hot and dry, and can be categorized as an arid subtropical zone, i.e., hot in summer and cold in winter. The temperature rises to as high as 53 °C in summer, while in winter it drops to low as 2 °C [43]. The average annual precipitation is 260 mm [44]. The most cultivated crops in the area are wheat and cotton and are grown within the delta plain of Sindh and in the annually inundated lands [35].

### 2.2. Geology and Hydrogeology

The area has a rough topography, and the land slope is 0.000095 from north to south. Soils are generally coarse in texture along former meander floodplains, whereas the soil texture is smoother and finer across the flooded cover plain. The area is covered mostly by meander floodplains and consists of bar deposits, channel in-fills and channel scars (Figure 1). Fine-textured soils constitute 36% of the area and the medium soil constitute 60%. Soil surface salinity occurs in small scattered patches throughout the research area [45]. The primary reason of prevalent salinization in the area is under-watering the crops, low cropping intensity, and seepage from canals and lateral channels. Chemical analysis reveals that almost 15% of the land under study is moderate-to-highly saline with saline-alkali land constituting over 5.5% of the total land mass [46].

Quaternary sediments are a dominant feature of the geology of the area, which can often be hundreds of meters thick. Lithological studies indicate that there is a 200 to 400 feet thick sandy layer present below the surface. They are well sorted, fine to medium micaceous sands with bands and lenses of silt and silty clay [35]. The deposits are extremely variable, and the variation in lithology is so intense that it is difficult to correlate the strata found in two adjacent boreholes. However, the relative percentage of sand and clay bands is remarkably constant over large areas. The depth to water table variates from 1.56 m to 11.93 m in this aquifer with an average depth of 4.53 m. The direction of groundwater flow is defined by the individual measurement of hydraulic head which are combined to obtain the contour maps of the water level (Figure 1). The hydraulic heads in the current study area range from 10m to 28 m. In upper part, the flow direction is more south westward, while in middle and lower part, the flow direction is west ward, more toward and along river Indus. Sand is generally the predominant material making a fairly unified, transmissive and non-artesian aquifer. Groundwater yields from these sediments are typically around 50–300 m^3^/h. The storage factor is about 0.2 and transmissibility is 2.58 cm^2^/s. Vertical permeabilities are low in comparison to lateral ones but are just enough to ensure that there is a transmission of recharge from layer to layer [47].

## 3. Materials and Methods

### 3.1. Sample Collection and Analysis

In order to assess the groundwater quality of the Rohri canal command area, a total of 59 groundwater samples were collected from wells, boreholes, and hand-pumps. Before sampling, wells were pumped for several minutes to avoid the effects of stagnant water. Samples were then stored in rinsed and properly washed 2.5 L glass bottles. These samples were then analyzed in the standard water quality laboratory of the Pakistan Council of Research in Water Resources (PCRWR). A pH meter and electrochemical analyzer (Hac 44600-00, Loveland, CO, USA) were used to measure the pH, electrical conductivity (EC), and total dissolved solid (TDS). An ultraviolet-visible (UV-VIS) spectrophotometer (Analytik Jena, Jena, Germany) was used to analyze samples for major anions, i.e., nitrate (NO_3_-N) and sulfate (SO_4_^2^^−^), while titration method was employed to analyze chloride (Cl^−^) and bicarbonates (HCO_3_^−^) [48]. To measure major cations like Na^+^, K^+^, and Fe^2+^, a Flame photometer (PFP7, Cambridgeshire, UK) was used. Volumetric titration with ethylene diamine tetra acetic acid (EDTA, 0.05 N) with <2% analytical error was used to analyze Ca^2+^ and Mg^2+^. Total alkalinity in the samples was measured by acid titration using methyl-orange. Atomic absorption spectrophotometer (AAS Vario 6 Analytik Jena, Jena, Germany) was used to determine arsenic.

To validate the quality of water analyses, charge balance error (CBE) was computed. Water samples having a higher concentration of cations show positive CBE, while negative CBE is credited to higher concentrations of anions [49]. CBE was computed by Equation (1);

(1) $$\mathrm{CBE}=\frac{\left[\Sigma \mathrm{cations}-\Sigma \mathrm{anions}\right]}{\left[\Sigma \mathrm{cations}+\Sigma \mathrm{anions}\right]}\times 100$$

where ionic concentrations are expressed in milliequivalent per liter (meq/L). According to the standard protocols, only those water samples were accepted that had less than ±5% CBE [50].

The Water Quality Index (WQI) values were computed to assess the suitability of groundwater for drinking purposes [51]. Three computing steps calculate WQI:

1. The first step involves the assignment of weight (*w_i_*) to each of the nine parameters (pH, TDS, Na, Mg, Ca, Cl, SO_4_, HCO_3,_ and K) based on their relative importance to the overall quality of groundwater (Table 1). Total dissolved solids, chloride and sulfate are given a maximum weight of 5 due to their significant role in assessment, while bicarbonate is given a minimum weight of 1 because of its insignificant importance. The other parameters (pH, Na, Mg, Ca and K) are assigned weights between 1 and 5 based on their significant role while assessing the evaluation of groundwater quality.
2. The second step involves the computation of relative weight (*W_i_*) of each parameter (Equation (2)).
  (2) $$W_{i}=\frac{w_{i}}{\sum_{i=1}^{n}w_{i}}$$
  where *W_i_* is the relative weight, *w_i_*_−_ is the weight of each parameter and *n* is the number of parameters.
3. The third step is based on computation of the quality rating scale (*qi*) for each parameter (Equation (3)).
  (3) $$q_{i}=\frac{C_{i}}{S_{i}}\times 100$$
  where *q_i_* is the quality rating, *C_i_* is the concentration of each parameter in mg/L, and *S_i_* is the WHO standard for each parameter in mg/L (Table 1).

The sub-index (*SI*) for each parameter is first calculated (Equation (4)) for the computation of WQI which requires the summation of sub-indices of all parameters in each sample (Equation (5)).

(4) $$SI_{i}=W_{i}\times q_{i}$$

(5) $$WQI=\overset{n}{\underset{i=1}{\sum }}SI_{i}$$

where *SI_i_* and *W_i_* is the sub-index and relative weight of the *i*th parameter, while *qi* is the rating based on the concentration of *i*th parameter.

For evaluating suitability for irrigation purposes, some commonly used indices like the sodium absorption ratio (SAR), residual sodium carbonate (RSC), sodium percentage (Na%), permeability index (PI), magnesium hazard (MH), Kelley’s ratio (KR) and potential salinity (PS) were computed [52]. Summary of the indices has been presented in Table 2.

### 3.2. Statistical Data Analysis

Statistical analyses play a pivotal role in interpreting the data set by representing various operations [53]. Regression along with Pearson correlation analysis was performed in SPSS (v23, SPSS Inc, Armonk, NY, USA) to delineate the relationships among water quality parameters. Piper diagram was prepared through Aqua-Chem (version 2010.1, Waterloo Hydrolgeologic, Kitchner, Ontario, Canada) to interpret hydrochemical facies. Saturation indices were calculated through geochemical modeling program PHREEQC (version 3.1) which determine the tendency of groundwater to dissolve or precipitate a particular mineral [54].

## 4. Results and Discussion

### 4.1. Chemical Characteristics of Groundwater

#### 4.1.1. Hydrochemical Parameters Statistics

Hydrochemical parameters were computed in accordance with WHO protocols [55]. Table 3 presents the statistical summary of analyzed hydrochemical parameters.

The value of turbidity ranges from 0 to 165 NTU (Nephelometric Turbidity Units) with a mean value of 7.82, which is relatively elevated. The high turbidity is due shallow and poorly built wells [56]. The pH value varies from 6.5–8.2 having a mean value of 7.41, depicting neutral to slightly alkaline nature of groundwater. The mean values of EC, total dissolved solids (TDS), and total hardness (TH) are 1570.97 µS/cm, 993.92 mg/L, and 421.12 mg/L respectively, and is higher than the WHO standard apart from TDS. Dissolution of minerals and soluble salts, and evaporation of shallow groundwater are the contributing factors of elevated salinity across the study area [46]. The concentration of Na^+^ and K^+^ ranges from 17 mg/L to 638 mg/L and 0 to 25 mg/L, with average values of 165.20 mg/L and 3.53 mg/L, respectively. Mainly, the dissolution of minerals containing Na^+^ and K^+^ with surrounding rocks leads to higher concentrations [57]. Ca^2+^ and Mg^2+^ vary from 14 mg/L to 220 mg/L and 10 mg/L to 175 mg/L respectively, with mean values of 76.46 mg/L and 55.41 mg/L, which are mainly due to carbonate minerals. The concentration of HCO_3_^−^ ranges from 59 mg/L to 950 mg/L, with an average concentration of 308.90 mg/L. SO_4_^2−^ and Cl^−^ varies from 20 mg/L to 600 mg/L and 16 mg/L to 779 mg/L respectively, with a mean values of 169.88 mg/L and 209.76 mg/L. Dissolution of gypsum, sulfate-bearing minerals and halite cause this rise in concentration [58]. According to the descriptive statistics of hydrochemical parameters, the abundance of cations is Na^+^ > Ca^2+^ > Mg^2+^, while the abundance of anions is HCO_3_^−^ > Cl^−^ > SO_4_^2−^.

Fluoride concentration varies from 0 mg/L to 2.0 mg/L with an average value of 0.42 mg/L, while most of the samples are in the permissible limits. Fluoride concentration in its extreme ranges portrays adverse effects on human health [59]. The value of NO_3_-N ranges from 0 mg/L to 9.90 mg/L having a mean value of 0.94 mg/L. The higher concentration of NO_3_-N is attributed to the excessive use of fertilizer and irrigation by wastewater but in the study area, all the samples have been found to be in the acceptable range. The concentration of Fe varies from 0 mg/L to 1 mg/L having a mean value of 0.09 mg/L.

Arsenic concentration in the research area ranges between 0 µg/L to 250 µg/L, while 15 (25.42%) samples were beyond the permissible range (10 µg/L). Elevated concentration of As in groundwater as a consequence of natural or anthropogenic sources has become a major environmental concern [60]. Climatic and geogenic sources have resulted in the varying concentration of As in groundwater, which is mainly because of moderately saline, alkalinity, and anoxic conditions [61]. It is agreed that groundwater contaminated with geogenic arsenic is more profound in alluvial aquifers [62,63]. The primary composition of these alluvial aquifers is sandstone, sand, silt, and gravel that remain in a flood plain or river channel over a longer period of time. The aquifer of the lower Indus is of similar composition [64]. As compared to other parts of the country, arsenic poisoning in the lower Indus is rather high [65], which is causing adverse effects on human health [66]. Natural mobilization of arsenic in groundwater is enhanced because of the pH-based reductive dissolution of iron hydroxide (FeOH) and competitive sorption of bicarbonate minerals in the presence of microorganisms along with evaporative enrichment. The anthropogenic causes of arsenic enrichment include water logging, coal-mining, and excessive use of pesticides [64].

#### 4.1.2. Hydrochemical Facies

Hydrochemical facies depict the overall scenario of the interaction of groundwater solutions within a lithological structure. They are quite beneficial in interpreting the groundwater transition and pattern of its flow [67]. Piper diagram [68] presents a comprehensive graphical representation of the hydrochemistry of samples and their hydrochemical regimes.

**Figure 2:**
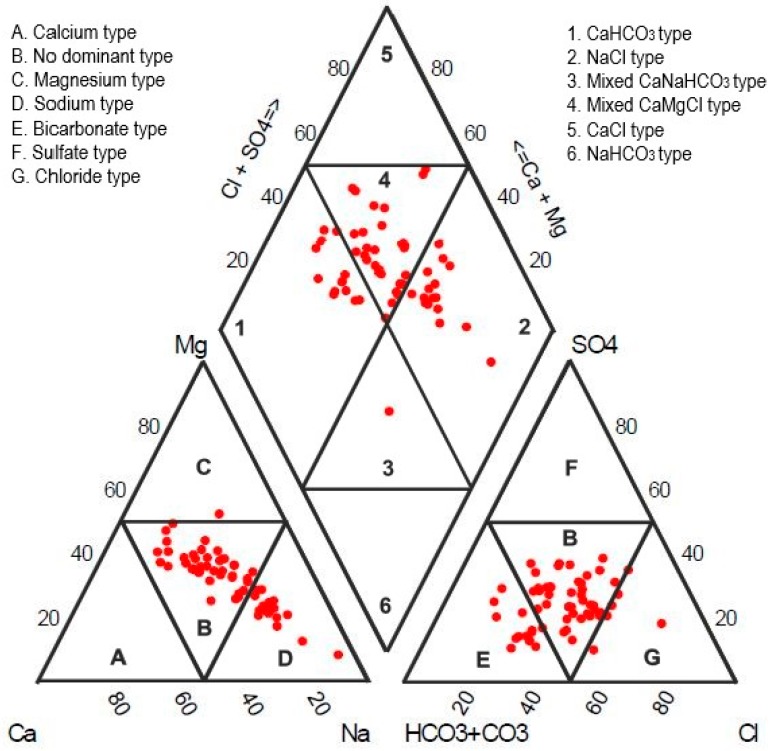
Piper plot for groundwater facies classification.

In order to elucidate the chemical differences, all the samples were plotted in a piper diagram (Figure 2). Most of the samples fall in Zone 4 (mixed CaMgCl type) and Zone 2 (NaCl type), where the rock–water interaction significantly interprets hydrochemical behavior, anthropogenic activities, interaction with the unsaturated zone, increased resident time, ion exchange and reverse ion exchange. Few samples lie in Zone 1 (CaHCO_3_ type), constituting fresh recharged water. With respect to cations, groundwater samples fall in Zone D (Na + K type) and Zone B (mixed type), depicting the prominence of silicate weathering and ion exchange. With respect to anions, most of the samples fall in Zone E (HCO_3_ type) and Zone B (mixed type), while few samples fall in Zone G (Cl type), representing the dominance of carbonate weathering and dissolution of evaporite while the dissolution of gypsum is minimal.

### 4.2. Sources of Major Ions and Hydrogeochemical Evolution

#### 4.2.1. Correlation Analysis

**Figure 3:**
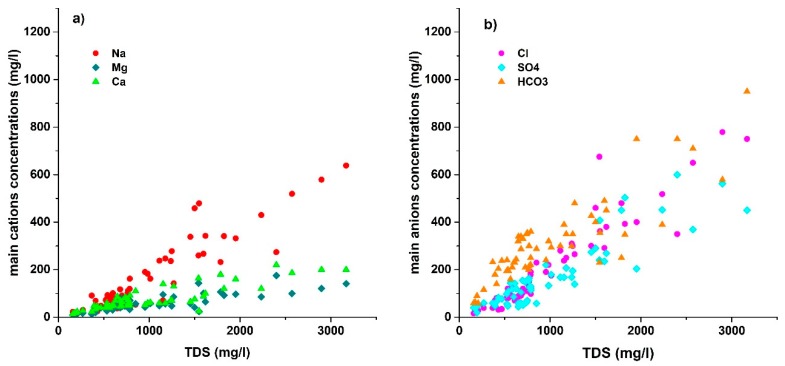
Scatter plot for (**a**) the main cations; (**b**) the main anions versus total dissolved solid (TDS).

According to the Pearson correlation matrix (Table 4), a strong correlation between Na^+^ and Cl^−^ (0.92), SO_4_^2−^ (0.85), Mg^2+^ (0.60), HCO_3_^−^ (0.75) exists, which depicts the derivation of Na^+^ from silicate weathering except the dissolution of halite and mirabilite, while the strong correlation of HCO_3_^−^ with Ca^2+^ (0.80) and Mg^2+^ (0.77) illustrate the dissolution of carbonate minerals (calcite and dolomite). A strong correlation between Ca^2+^ and SO_4_^2−^ (0.73) suggests the dissolution of gypsum. The association between TDS and major constituent ions is beneficial in explaining the hydrochemical processes. TDS has been found to be strongly correlated with Na^+^ (0.92), Ca^2+^ (0.86), Cl^−^ (0.94), Mg^2+^ (0.84), SO_4_^2−^ (0.90) and HCO_3_^−^ (0.86). All the ionic concentrations increase with the increase in TDS value (Figure 3).

#### 4.2.2. Silicate Weathering

**Figure 4:**
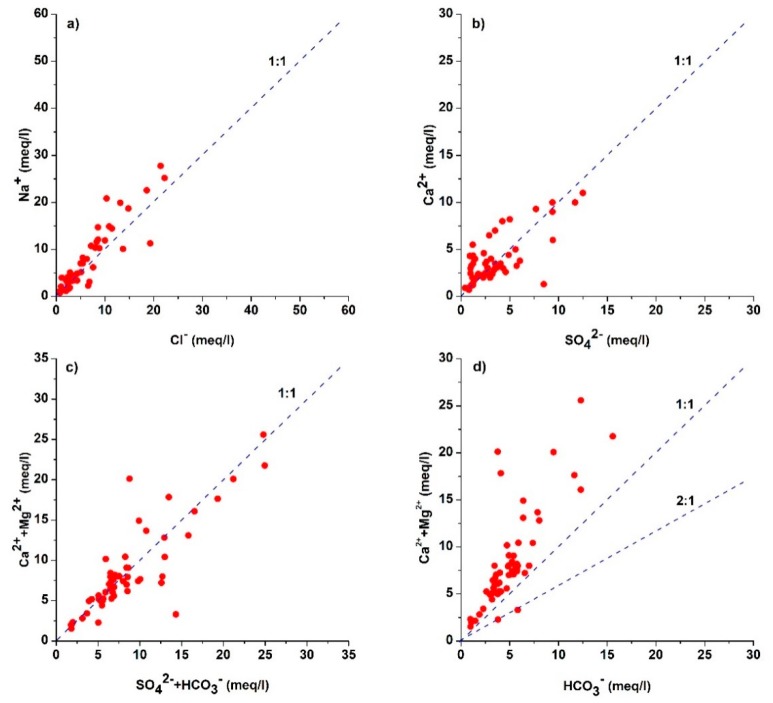
Relationship between ions (**a**) Na^+^ versus Cl^−^ (**b**) Na^+^ versus TZ^+^ (**c)** Ca^2+^ + Mg^2+^ versus SO_4_^2−^ + HCO_3_^−^ (**d)** Ca^2+^ versus SO_4_^2^.

The contribution of silicate weathering plays a pivotal role in controlling the major ion chemistry of groundwater [69]. It is hard to quantify the silicate weathering products due to the incongruent nature of degradation of silicates, which yields a variety of solid phases (mostly clays) with other dissolved species [70]. Sodium which is the most abundant cation in the study area is derived from silicate weathering and by the dissolution of halite. If halite dissolution is the sole source of sodium (Equation 6), then the molar ratio of Na^+^/Cl^−^ would be equal to one, while the deviation from 1:1 line (Figure 4a) is attributed to the contribution of silicate weathering and ion exchange, e.g., the dissolution of albite (Equation (7)) might be responsible for the increment of Na^+^ in groundwater.

**Figure 5:**
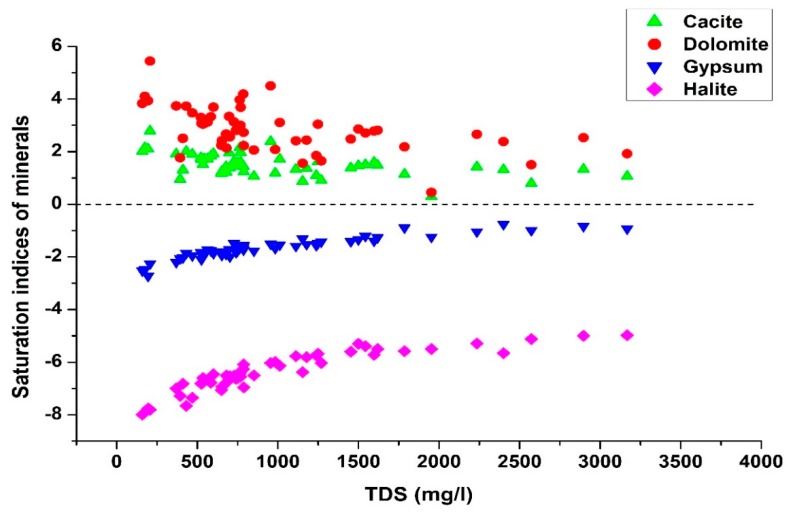
Variation of saturation indices of selected minerals.

This fact is well supported by the saturation index of halite, which is under saturated with respect to water (Figure 5).

(6) $$\mathrm{NaCl}\to {\mathrm{Na}}^{+}+{\mathrm{Cl}}^{-}.$$

(7) $$2{\mathrm{NaAlSi}}_{3}O_{8}+2{\mathrm{CO}}_{2}+11H_{2}O\to {\mathrm{Al}}_{2}{\mathrm{Si}}_{2}O_{5}{\left(\mathrm{OH}\right)}_{4}+4H_{4}{\mathrm{SiO}}_{4}+2{\mathrm{Na}}^{+}+2{\mathrm{HCO}}_{3}^{-}$$

**Figure 6:**
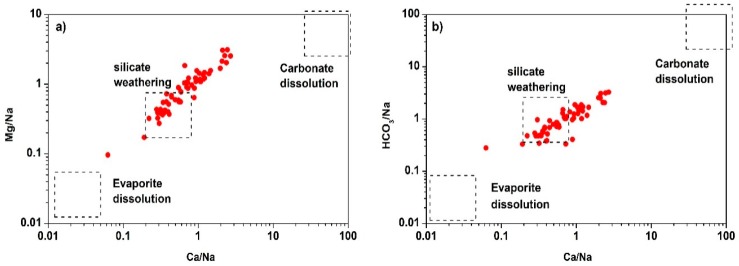
Plots of (**a**) Na-normalized Ca^2+^ versus Mg^2+^ (**b**) Na-normalized Ca2+versus HCO_3_^−^.

The influence of silicate weathering and carbonate dissolution is further illustrated by Na-normalized Ca^2+^ versus Mg^2+^ plot (Figure 6a) and Na-normalized Ca^2+^ versus HCO_3_^−^ plot (Figure 6b). The Na-normalized Ca^2+^ versus Mg^2+^ plot shows that most of the Mg^2+^ is derived from carbonate dissolution except the silicate weathering, while Na-normalized Ca^2+^ versus HCO_3_^−^ plot demonstrates that samples tend to fall close to silicate weathering rather than carbonate and evaporite dissolution [71].

If silicate weathering is a prominent source of sodium, then the most abundant anion should be HCO_3_^−^ [72]. The abundance of bicarbonate among anions justifies this statement. The estimation of the ratio between Na^+^ and total cations (TZ^+^) would be helpful in understanding silicate weathering (Figure 4b). The majority of the samples fall near Na^+^ = 0.5 TZ^+^ line, which indicates the contribution of silicate weathering in the concentration of sodium in groundwater.

The contribution of dissolution of carbonates (calcite and dolomite) and sulfates (gypsum) is illustrated by the association between Ca^2+^ + Mg^2+^ and SO_4_^2−^ + HCO_3_^−^ (Figure 4c). Samples falling on equiline (Ca^2+^ + Mg^2+^ and SO_4_^2−^ + HCO_3_^−^) indicate dissolution of carbonates and sulfate as the sole source of Ca^2+^, Mg^2+,^ and SO_4_^2−^ in groundwater (Equation 8, 9), while samples lying above the 1:1 line indicate less contribution of Ca^2+^ and Mg^2+^ by the dissolution of carbonates. The oversaturation of calcite and dolomite with respect to water indicated by the saturation indices of these minerals proves this statement (Figure 5).

(8) $${\mathrm{CaCO}}_{3}+{\mathrm{CO}}_{2}+H_{2}O\to {\mathrm{Ca}}^{2+}+2{\mathrm{HCO}}_{3}^{-}$$

(9) $$\mathrm{CaMg}{\left({\mathrm{CO}}_{3}\right)}_{2}+2{\mathrm{CO}}_{2}+2H_{2}O\to {\mathrm{Mg}}^{2+}+{\mathrm{Ca}}^{2+}+4{\mathrm{HCO}}_{3}^{-}$$

According to Figure 4d, samples falling along the 1:1 line indicate the dissolution of gypsum as a contributor of Ca^2+^ and SO_4_^2−^ ions in groundwater (Equation (10)). Moreover, under-saturation of gypsum with respect to water (Figure 5) allows it to dissolve in groundwater. However, samples falling below the equiline demonstrate the contribution of SO_4_^2−^ by the dissolution of Glauber’s salt (NaSO_4_·10 H_2_O) (Equation (11)), which also elevates the concentrations of Na^+^ ions in the groundwater.

(10) $${\mathrm{CaSO}}_{4}\cdot 2H_{2}O\to {\mathrm{Ca}}^{2+}+{\mathrm{SO}}_{4}^{2-}+2H_{2}O$$

(11) $${\mathrm{Na}}_{2}{\mathrm{SO}}_{4}\cdot 10H_{2}O\to 2{\mathrm{Na}}^{+}+{\mathrm{SO}}_{4}^{2-}+10H_{2}O$$

#### 4.2.3. Ion Exchange

Chloro-alkaline indices (CAI-1 and CAI-2) proposed by Schoeller [73] provide information about the ion exchange reactions, which have significant impacts on groundwater chemistry and its evolution [74,75]. The following formula can calculate the two indices:

(12) $$\mathrm{CAI}-1=\frac{{\mathrm{Cl}}^{-}-({\mathrm{Na}}^{+}+K^{+})}{{\mathrm{Cl}}^{-}}$$

(13) $$\mathrm{CAI}-2=\frac{{\mathrm{Cl}}^{-}-\left({\mathrm{Na}}^{+}+K^{+}\right)}{{\mathrm{HCO}}_{3}^{-}+{\mathrm{SO}}_{4}^{2-}+{\mathrm{CO}}_{3}^{2-}+{\mathrm{NO}}_{3}^{-}}$$

where concentrations are in meq/L.

The direct ion exchange occurs when both indices give positive values expressed by Equation (14), while Equation (15) indicates the reverse ion exchange as a result of negative values of CAI-1 and CAI-2.

(14) $$2{\mathrm{Na}}^{+}+{\mathrm{CaX}}_{2}\to {\mathrm{Ca}}^{2+}+2\mathrm{NaX}$$

(15) $${\mathrm{Ca}}^{2+}+2\mathrm{NaX}\to 2{\mathrm{Na}}^{+}+{\mathrm{CaX}}_{2}$$

where soil exchange is expressed by X [76].

**Figure 7:**
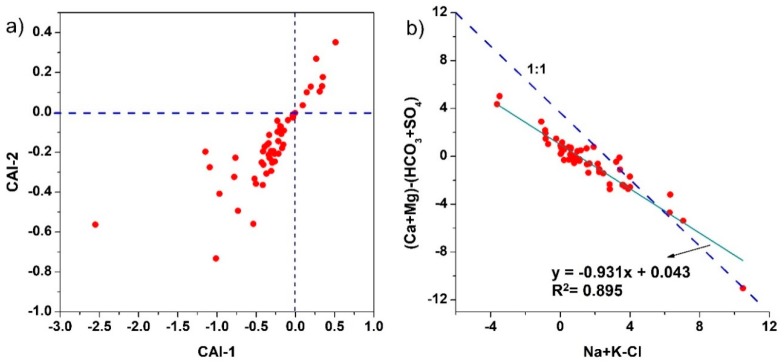
Plots showing (**a**) CAI-1 versus CAI-2; (**b**) (Ca^2+^ + Mg^2+^) − (HCO_3_^−^ + SO_4_^2−^) versus (Na^+^ + K^+^) − Cl^−.^

The possible role of ion exchange in hydrochemistry and evolution of groundwater can be studied by bivariate diagram between (Figure 7b) (Na^+^ + Cl^−^ + K^+^) and [(Ca^2+^ + Mg^2+^) – (SO_4_^2−^ + HCO_3_^−^)] [77]. (Na^+^ + Cl^−^ + K^+^) indicates the increment of Na^+^ in the system by the processes other than halite dissolution, while [(Ca^2+^ + Mg^2+^) – (SO_4_^2−^ + HCO_3_^−^)] represents the increment of cations Ca^2+^ and Mg^2+^ that are credited to processes excluding carbonate or silicate weathering (dissolution of gypsum, calcite, and dolomite) [78]. A linear relation between the parameters with slope equal to −1 indicates the significance of ion exchange as an important factor controlling the groundwater chemistry and its evolution [79]. According to Figure 7b, the relationship between parameters shows linearity with a slope value of −0.931 (Equation (16)), which is very close to the theoretical value of −1, indicating the ion exchange between Na^+^, Ca^2+^, and Mg^2+^.

y = −0.931x +0.043 (R^2^ = 0.895) (16)

Most of the samples are plotted in the lower left part of Figure 7a, indicating reverse ion exchange expressed by Equation (11), which leads to the increment of Na^+^ and decrement of Ca^2+^ in groundwater. However, few samples fall in the upper right part, which signify direct ion exchange (Equation (10)).

#### 4.2.4. Groundwater Chemistry Formation Mechanism

**Figure 8:**
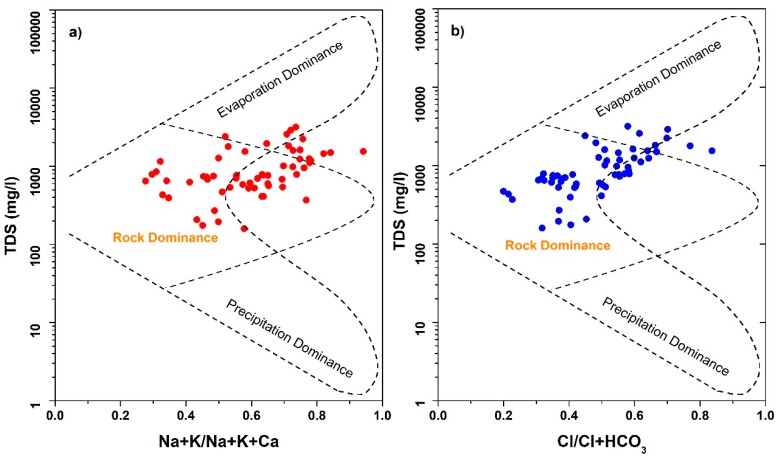
Gibbs plot showing major processes controlling groundwater chemistry.

Groundwater resource development and quality protection require a better understanding of the key factors governing groundwater chemistry [75]. Gibbs (1970) proposed his well-known Gibbs diagram for studying groundwater chemistry formation mechanism. The two sub diagrams (Figure 8a,b) include relationship of TDS with weight ratio of (Na^+^ + K^+^) versus (Na^+^ + K^+^ + Ca^2+^) and TDS with weight ratio of Cl^−^ versus (Cl^−^ + HCO_3_^−^). According to Gibbs’ diagram, three factors influence the groundwater chemistry, which include rock dominance, evaporation dominance and precipitation dominance [80,81]. According to Figure 8, most of the samples lie in the rock dominance zone, indicating that rock weathering is the primary source that controls the groundwater chemistry and its evolution. Parent rock weathering facilitates the process by which dissolvable salts and minerals become incorporated with groundwater. Moreover, long residence time of rock–water interaction also aids the mineral dissolution [57]. Figure 8 also shows that few samples fall in evaporation dominance zone. The influence of evaporation is attributed to the shallow groundwater depth (typically less than 3 m), which results in intense evaporation in the alluvial plain.

### 4.3. Groundwater Quality Assessment

The suitability of groundwater for drinking and irrigation is assessed on the basis of hydrochemical analysis data.

#### 4.3.1. Assessment of Groundwater Quality for Drinking Purposes

WQI is one of the most comprehensive ways to address the overall quality of groundwater [82]. The composite influence of groundwater chemical parameters on overall groundwater quality is provided by the WQI, which is calculated by considering the standards recommended for drinking purposes by the WHO [55].

**Figure 9:**
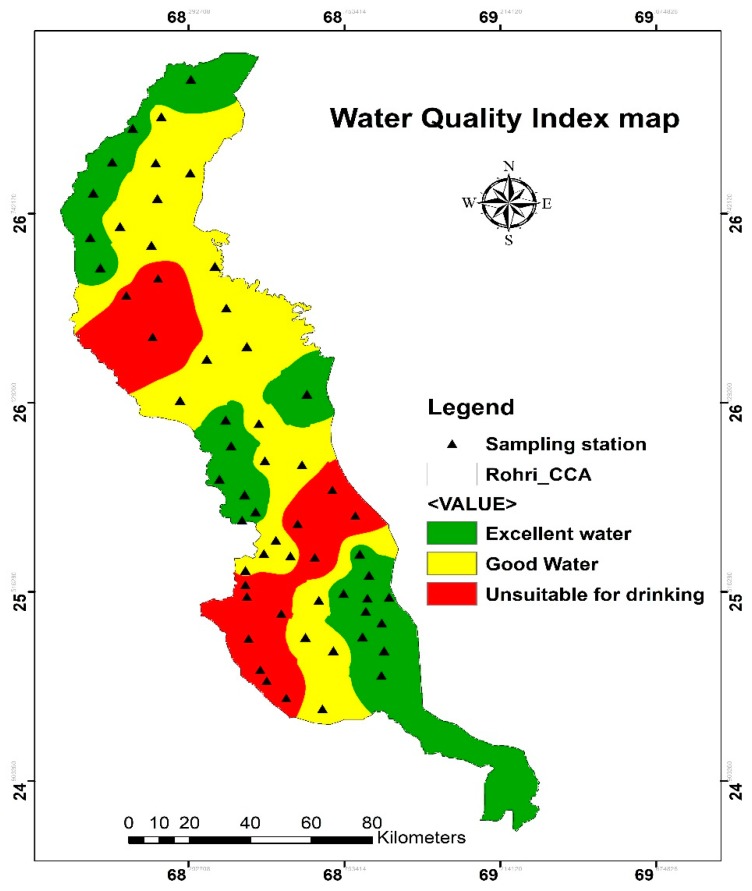
Water Quality Index map.

WQI values are classified into five categories: excellent (<50), good (>50), poor (>100), very poor (>200) and water unsuitable for drinking (>300). Table 5 illustrated the categories of groundwater quality according to WQI while the spatial distribution of water types is presented in Figure 9. The WQI values range from 35.15 to 231.46. According to Table 5, 9 samples (15.25%) are considered “excellent”, 33 samples (55.93%) as “good” while 17 samples (28.82%) were classified as “water unsuitable for drinking”.

#### 4.3.2. Assessment of Groundwater Quality for Irrigation Purposes

The suitability of groundwater for irrigation is assessed through United States Salinity Laboratory (USSL) diagram (1954) and Wilcox diagram [83] along with some indices, such as SAR, RSC, %Na, PI, MH, KR and PS. A statistical summary of irrigation quality indices of groundwater samples is presented in Table 6.

**Figure 10:**
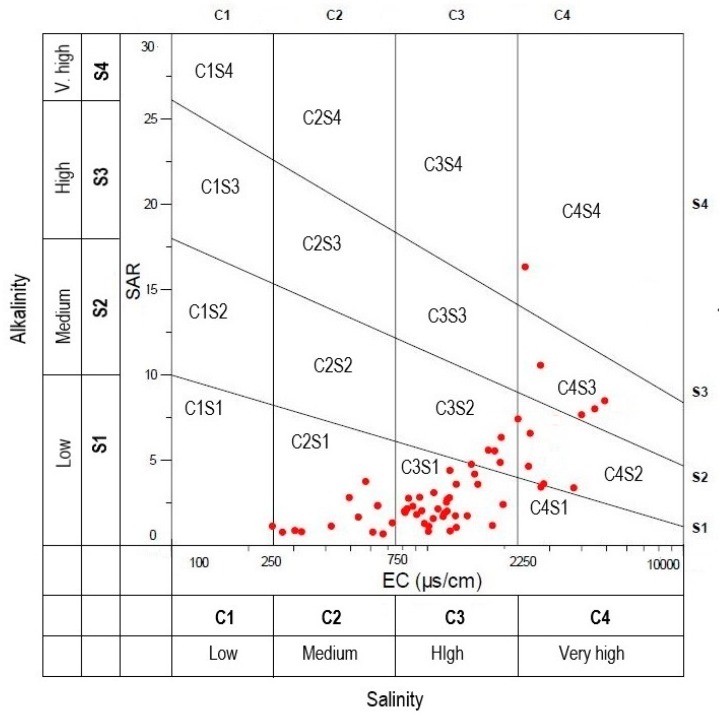
United States Salinity Laboratory (USSL) diagram for irrigation water classification (USSL 1954).

SAR or Alkali hazard is an important tool to determine the groundwater suitability for irrigation purposes. Osmotic activity is reduced by higher salinity which hinders water to reach the branches and leaves of the plants, leading to their inferior production [84]. According to SAR, groundwater is classified as low (SAR < 10), medium (10 < SAR ≤ 18), high (18 < SAR ≤ 26) and very high (SAR > 26) sodium hazard. In the current study, the value of SAR ranges from 0.65 to 16.21, with an average of 3.41 depicting a low or medium sodium hazard. The USSL diagram (Figure 10) shows that 44 samples (73.78%) fall in C_2_S_1_ and C_3_S_1_ categories which can be used for irrigation purpose with little harm of Na^+^ exchange. Four samples belong to C_3_S_2_ category, six samples to C_4_S_2_ category, four samples to C_4_S_3_ category, while only one samples fall in C_4_S_4_ category. Water falling under these categories cannot be recommended for any agricultural practices.

RSC is an important index for the evaluation of groundwater suitability for irrigation. It can be estimated by subtracting alkaline earth metals (Ca^2+^ + Mg^2+^) from carbonates and bicarbonates (CO_3_^2−^ + HCO_3_^−^). Adsorption of sodium in soil is attributed to the high value of RSC [85]. Groundwater is considered unsuitable if RSC value exceeds 2.5. According to RSC, 98.30% samples of the present study have been found to be suitable for irrigation purposes.

**Figure 11:**
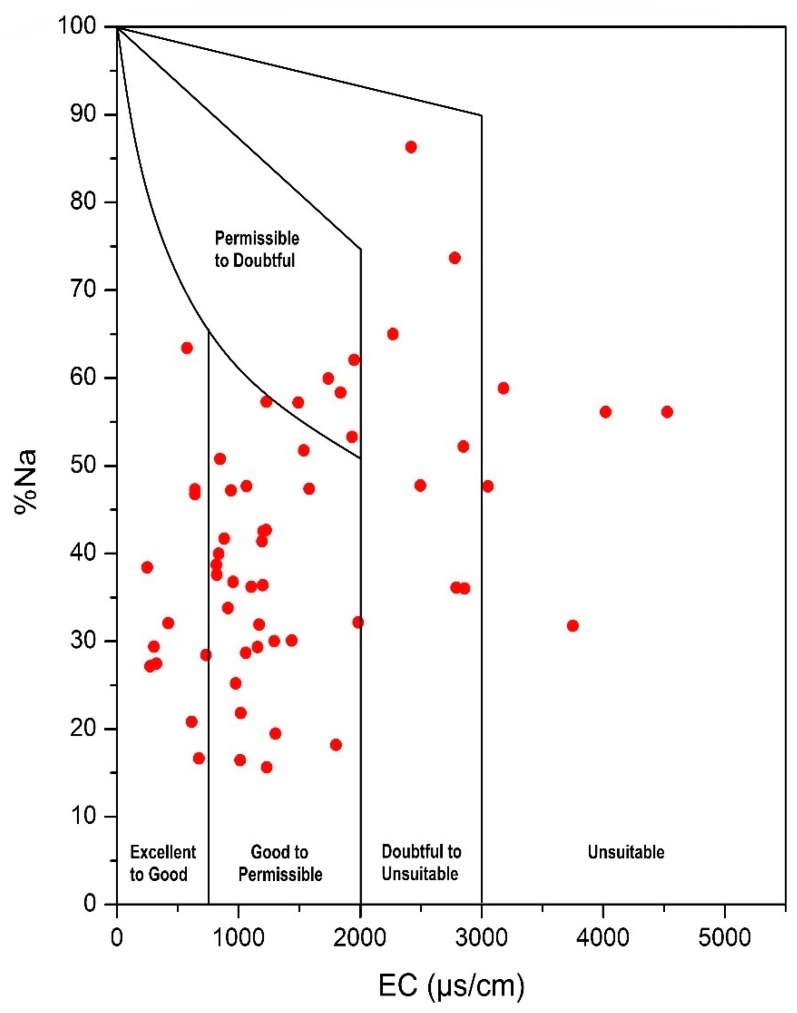
Plot of percentage sodium (%Na) versus EC (Wilcox 1948).

Irrigation water with high sodium percentage reduces the permeability of the soil, which consequently affects the plant growth, thus making it an important index for the evaluation of groundwater on agricultural scale [86]. Groundwater having less than 60% Na is considered suitable for irrigation use. %Na value ranges from 15.64 to 86.32 having a mean value of 41.70. 91.52% samples fall in excellent to permissible limits. According to Wilcox diagram (Figure 11), 42 samples (71.19%) lie in excellent to permissible zone, five samples in permissible to doubtful, while seven samples in doubtful to unsuitable and only five samples are in unsuitable zone.

The influence of ion contents in groundwater on the permeability of the soil is evaluated by Permeability Index (PI) proposed by Doneen [87]. Based on PI, values less than 25 are considered unsuitable for irrigation. In the current study, all samples have been found suitable for irrigation on PI basis.

Another tool for assessing the agricultural suitability of groundwater is MH, proposed by Szabolcs and Darab [88]. Sodication in the soils increases with the subsequent increment of Mg^2+^ content relative to Ca^2+^, which consequently results in the dispersion of clay particles hence damaging the soil structure by decreasing the hydraulic conductivity. MH value less than 50 is considered suitable for irrigation. Table 6 shows that 69.49% of samples have been found in the suitable range.

KR is another important indicator for the evaluation of groundwater for agricultural suitability proposed by Kelly [89]. A Kelly’s ratio value greater than 1 indicates excess level of sodium in groundwater, while less than 1 Kelly’s ratio is considered suitable for irrigation [90]. In the current study, as per KR, 42 samples (71.19%) fall in the suitable range for irrigation purposes.

The agricultural suitability of groundwater is also assessed by PS which is defined as the chloride concentration plus half of the sulfate concentration. Doneen [91] proposed that agricultural suitability is not dependent on the concentration of soluble salts. Every successive irrigation helps in the precipitation and accumulation of low soluble salts, whereas the salinity of the soil is enhanced by the concentration of highly soluble salts. Groundwater is considered suitable, when PS values are less than 10. For the present study, PS values show that 43 samples (72.89%) are suitable for irrigation purposes.

## 5. Conclusions

In order to carry out the current study, an amalgamation of statistical analyses, graphical techniques and hydrochemical modeling were used for comprehensive understanding of the groundwater chemistry, its evolution and suitability for drinking and agricultural purposes in the delimited area of research.

Statistical analyses demonstrate that the abundance of cations is in the order: Na^+^ > Ca^2+^ > Mg^2+^ > K^+^, while the abundance of anions is in the order: HCO_3_^−^ > Cl^−^ > SO_4_^2−^. The abundance of Na^+^ and HCO_3_^−^ proves that silicate weathering is the eminent phenomenon controlling the major ion chemistry of the groundwater in the research area. This is also supported by the correlation analysis and graphical relationships between ions that reveal that ion exchange and rock weathering—like the dissolution of halite, albite, and dissolution of carbonate minerals—are important rock–water interactions, which govern the evolution of groundwater chemistry. Furthermore, rock dominance has been found as the key natural factor governing the groundwater evolution as depicted by the Gibbs diagrams, while few samples falling in the evaporation dominance propose the influence of evaporation in shallow groundwater depth zone. Hydrochemical facies are predominantly of mixed CaMgCl and Na-Cl type with few samples of Ca-HCO_3_ type, constituting fresh recharged water. Saturation indices computed by geochemical modelling indicate that the aqueous phase is under-saturated with respect to evaporites like halite and gypsum (negative values), while there is equilibrium to the over-saturated phase with respect to carbonates like calcite and dolomite (positive values).

Computed values of WQI shows that 71.18% samples are safe for drinking purpose while 28.82% fall in unsuitable range. The contributing factors towards the aforementioned range of groundwater samples include dissolution process and effective leaching of rock salt and gypsum-bearing rock formations. The majority of samples have been found suitable for irrigation purposes as per the USSL diagram, Wilcox diagram and other agricultural indices.

## Figures and Tables

**Table 1:** Weight and relative weight of each chemical parameter.

Chemical Parameters	WHO Standard (mg/L) (S_i_)	Weight (w_i_)	Relative Weight (W_i_)
pH	6.5–8.5	3	0.097
TDS	1000	5	0.161
Na	200	4	0.129
Mg	150	3	0.097
Ca	200	3	0.097
Cl	250	5	0.161
SO_4_	250	5	0.161
HCO_3_	250	1	0.032
K	12	2	0.065
		$\Sigma w_{i}=31$	$\Sigma W_{i}=1$

**Table 2:** Summary of water quality indices for irrigation.

Indices	Acronym	Formula
Sodium absorption ratio	SAR	${\mathrm{Na}}^{+}/\sqrt{\left({\mathrm{Ca}}^{2+}+{\mathrm{Mg}}^{2+}\right)/2}$
Residual sodium carbonate	RSC	$({\mathrm{CO}}_{3}{}^{2-}+{\mathrm{HCO}}_{3}{}^{\_})-\left({\mathrm{Ca}}^{2+}+{\mathrm{Mg}}^{2+}\right)$
Sodium percentage	%Na	$[({\mathrm{Na}}^{+}+K^{+})/\left({\mathrm{Na}}^{+}+K^{+}+{\;\mathrm{Ca}}^{2+}+{\mathrm{Mg}}^{2+}\right)]\times 100$
Permeability index	PI	$[({\mathrm{Na}}^{+}+\sqrt{{\mathrm{HCO}}_{3}{}^{\_}})/({\mathrm{Ca}}^{2+}+{\mathrm{Mg}}^{2+}$ + ${\mathrm{Na}}^{+})]\times 100$
Magnesium hazard	MH	$\left[\left({\mathrm{Mg}}^{2+}\right)/\left({\mathrm{Ca}}^{+2}+{\mathrm{Mg}}^{2+}\right)\right]\times 100$
Kelly’s ratio	KR	$\left[({\mathrm{Na}}^{+})\;/\left({\mathrm{Ca}}^{2+}+{\mathrm{Mg}}^{2+}\right)\right]$
Potential salinity	PS	${\mathrm{Cl}}^{-}+\sqrt{{\mathrm{SO}}_{4}{}^{2-}}$

**Table 3:** Statistical analyses of chemical parameters (units of all parameters are mg/L, except pH, electrical conductivity (EC) µS/cm, Turbidity NTU and arsenic µg/L).

Parameters	Minimum	Maximum	Mean	Standard Deviation	WHO Standards	NSBL *	NSBL %
Turbidity	0	165	7.82	27.29	5	10	16.95
pH	6.70	8.20	7.41	0.33	6.5–8.5	0	0
EC	249	4950	1570.97	1061.90	1000	39	66.10
TDS	159	3168	993.92	677.51	1000	21	35.59
TH	75	1270	421.12	252.98	300	40	67.80
Alkalinity	0	19	5.69	3.69	-		0
Na^+^	17	638	165.20	151.79	200	18	30.51
K^+^	0	25	3.53	5.15	12	4	6.78
Mg^2+^	10	175	55.41	34.31	150	1	1.69
Ca^2+^	14	220	76.46	49.65	200	3	5.08
Fe	0	1	0.09	0.19	0.3	2	3.39
F^−^	0	2	0.42	0.50	1.5	4	6.78
Cl^−^	16	779	209.76	186.32	250	19	32.20
SO_4_^2−^	20	600	169.88	140.07	250	11	18.64
HCO_3_^−^	59	950	308.90	170.29	250	33	55.93
NO_3_-N	0	9.90	0.94	1.60	10	0	0
As	0	250	21.95	48.31	10	15	25.42

* Number of samples beyond (WHO) limits.

**Table 4:** Correlation coefficient matrix of major physiochemical parameters.

Parameter	pH	EC	TDS	TH	Na^+^	K^+^	Mg^2+^	Ca^2+^	Cl^−^	SO_4_^2−^	HCO_3_^−^
**pH**	1										
**EC**	−0.176	1									
**TDS**	−0.167	0.995 *	1								
**TH**	−0.227	0.891 *	0.888 *	1							
**Na^+^**	−0.082	0.925 *	0.923 *	0.659 *	1						
**K^+^**	0.086	0.140	0.128	0.152	0.100	1					
**Mg^2+^**	−0.301	0.847 *	0.838 *	0.971 *	0.602 *	0.138	1				
**Ca^2+^**	−0.166	0.863 *	0.859 *	0.977 *	0.626 *	0.161	0.924 *	1			
**Cl^−^**	−0.080	0.955 *	0.942 *	0.812 *	0.917 *	0.130	0.771 *	0.811 *	1		
**SO_4_^2−^**	−0.190	0.899 *	0.904 *	0.790 *	0.849 *	0.091	0.753 *	0.730 *	0.833 *	1	
**HCO_3_^−^**	−0.215	0.858 *	0.865 *	0.830 *	0.746 *	0.145	0.774 *	0.800 *	0.716 *	0.659 *	1

* Correlation is significant at the 0.01 level (2-tailed). **Bold =** strong correlation (>0.90).

**Table 5:** Classification of groundwater quality according to the Water Quality Index (WQI).

WQI	Water Type	No. of Samples	Percentage of Samples
<50	Excellent water	9	15.25
50–100	Good water	33	55.93
>100	Unsuitable for drinking	17	28.82

**Table 6:** Statistical summary of irrigation quality indices of groundwater samples.

Indices	Minimum	Maximum	Mean	SD	Permissible Limit	Unsuitable Samples	Suitable Samples %
SAR	0.65	16.21	3.41	0.37	≤18	-	100
RSC	−16.35	2.52	−3.38	0.45	≤2.5	1	98.30
%Na	15.64	86.32	41.70	1.98	≤60	5	91.52
PI	31.19	96.32	59.67	1.76	>25	-	100
MH	33.94	65.53	46.83	0.75	≤50	18	69.49
KR	0.18	6.31	0.89	0.11	≤1	17	71.19
PS	1.37	25.68	7.74	0.77	≤10	16	72.89
